# Facial Expression Recognition Robust to Occlusion and to Intra-Similarity Problem Using Relevant Subsampling

**DOI:** 10.3390/s23052619

**Published:** 2023-02-27

**Authors:** Jieun Kim, Deokwoo Lee

**Affiliations:** Department of Computer Engineering, Keimyung University, Daegu 42601, Republic of Korea

**Keywords:** facial expression recognition, spatial transformation network, attention mechanism, triplet loss function, intra-similarity problem

## Abstract

This paper proposes facial expression recognition (FER) with the wild data set. In particular, this paper chiefly deals with two issues, occlusion and intra-similarity problems. The attention mechanism enables one to use the most relevant areas of facial images for specific expressions, and the triplet loss function solves the intra-similarity problem that sometimes fails to aggregate the same expression from different faces and vice versa. The proposed approach for the FER is robust to occlusion, and it uses a spatial transformer network (STN) with an attention mechanism to utilize specific facial region that dominantly contributes (or that is the most relevant) to particular facial expressions, e.g., anger, contempt, disgust, fear, joy, sadness, and surprise. In addition, the STN model is connected to the triplet loss function to improve the recognition rate which outperforms the existing approaches that employ cross-entropy or other approaches using only deep neural networks or classical methods. The triplet loss module alleviates limitations of the intra-similarity problem, leading to further improvement of the classification. Experimental results are provided to substantiate the proposed approach for FER, and the result outperforms the recognition rate in more practical cases, e.g., occlusion. The quantitative result provides FER results with more than 2.09% higher accuracy compared to the existing FER results in CK+ data sets and 0.48% higher than the accuracy of the results with the modified ResNet model in the FER2013 data set.

## 1. Introduction

Recognition problems have always been issues in computer vision and pattern recognition. In particular, face or facial expression recognition is considered the most widely explored topic in research and industrial fields. Computer vision, pattern recognition, and imaging-related technologies have achieved impressive performance from quantitative and qualitative perspectives in recent years with the appearance of end-to-end learning frameworks, such as deep neural network models. Among numerous practical applications of computer vision and pattern recognition, image-based, automatic, and intelligent facial expressions are considered one of the most popular topics because facial expression conveys emotional states and can play a key role in detecting, analyzing, and predicting emotional or behavioral states. In facial expression recognition (FER), researchers have usually dealt with discrete facial expressions, such as happiness, surprise, neutral, sadness, fear, disgust, and anger [1]. Thus, FER aims to achieve accurate classification among different facial expressions, i.e., maximize inter-class distance and minimize intra-class distance.

Over a few decades, numerous approaches have been proposed, and they are categorized into two groups, conventional ones, and deep-learning-based ones. Similar to a field of object recognition, conventional FER is usually composed of three major steps: (1) preprocessing of an image containing a facial image followed by detection of the face region, (2) extracting features of a face, and (3) classification and recognition of expressions. From a technological perspective, FER is similar to face recognition (FR) [2], but FER is different from FR in that FER chiefly deals with the seven target expressions mentioned above. Moreover, facial expressions play a more important role in human communication or other interactions between human–machine and human–human. FR usually plays a key role in human identification or authentication rather than interaction activities.

### 1.1. Traditional Methods

Pre-processing in FER requires reliable quality of image data so that feature extraction and face detection can be accurately achieved. Noise reduction (or removal) is carried out before detecting the region of interest, e.g., face detection. Various types of filters usually categorize in low pass filters, such as Gaussian filter, Laplacian of Gaussian (LOG) filter or bilateral filter. Histogram data are sometimes utilized to enhance image quality, e.g., histogram stretching, histogram equalization, etc. If a facial image contains illumination, varying pose, or occlusion, more complex preprocessing techniques are required [3]. Furthermore, a face image can be acquired using various types of sensors or using a combination of multiple sensors, e.g., fusion of RGB and IR (infrared red) sensors, leading to increasing complexity of algorithms. In the course of recognition, it can successfully begin with accurate detection of the region of interest (ROI). In FER, accurate detection of the face region needs to be carried out before performing expression recognition. Human face detection has also been one of the most important processes in face recognition, expression recognition, gesture recognition, etc.

In conventional approaches of FER or FR (without using deep learning), tremendous work for face detection was proposed [4], and it can be categorized into feature-based and image-based approaches. The former includes active shape model (ASM), low-level analysis (color, motion, or edge-based analysis), and feature analysis (Viola Jones detector, AdaBoost, local binary pattern, Gabor feature-based method, constellation method, etc.). The latter includes more recent approaches that use training and test data sets to perform the matching procedure for the detection (neural network, principal component analysis (PCA), and support vector machine (SVM)).

The image-based approach also contains a sub-space-based method and statistical approach (PCA and SBVM are also included in this category). In feature-based face detection, accurate feature extraction (invariant feature points) is desired, while the image-based approach achieves accuracy and computational efficiency by performing dimensionality reduction. Once the region of a face is detected, feature extraction is carried out. Accurate feature extraction is crucial for diverse applications of image processing and computer vision. Almost all imaging and vision technologies require highly accurate feature extraction results. Feature extraction has been one of the most significant contributions to FER, and there have been extensive research activities to propose accurate feature extraction algorithms. In FER or FR, feature extraction and face detection are very closely related and highly correlated, and some of the algorithms are overlapped. In addition, extracted landmarks are important in many facial tasks [5,6]. In feature extraction of a facial image, applying proper spatial filters to a facial image is a very basic and simple approach. The Gabor filter, local binary pattern (LBP), scale-invariant feature transform (SIFT), speed-up robust features (SURF), and histograms of oriented gradients (HOG) are the most popularly used ones. Encoding based on a code-book is another approach for feature extraction, composed of a training phase and an encoding phase. K-means algorithm, Gaussian mixture model (GMM), and Fisher Vector (FV) are encoding-based approaches. Spatial pooling and holistic encoding also play roles in feature extraction.

Classification is the final stage for recognition. In the recognition, inter-class distance is to be maximized, while intra-class distance is to be minimized. Numerous conventional approaches have been used for recognition recently, e.g., Hausdorff distance (HD), Euclidean distance (ED), SVM, PCA, hidden Markov model (HMM), hidden conditional random fields (HCRF), etc. [7,8,9,10,11].

### 1.2. Deep-Learning-Based Methods

Although tremendous efforts have been made to improve performance of traditional FER from qualitative and quantitative perspectives, it still lacks recognition accuracy when used in an uncontrolled experimental environment, with images that belong to a wild setting, or with unrefined input images. Similar to other image processing, computer vision, and pattern recognition problems, recent FER has shown remarkable improvements by employing deep learning models [12]. Deep-learning-based FER uses a deep neural network that has various types of structures each of which has its strengths. Proper selection of the model can significantly improve the performance of face detection, feature extraction, and classification for the recognition. A deep neural network (DNN), with a sufficiently large amount of data, provides an end-to-end framework for FER tasks. The recent state-of-the-art approaches have verified the advantages in the fields of visual object recognition, pose estimation, depth estimation and others [13]. Deep-learning-based FER aims at classifying facial expressions using a single image or sequence of images, and the neural network structures learn characteristics or information contained in image data sets. Even if not under controlled experimental environments, deep-learning-based FER provides accurate and reliable recognition results. In other words, in contrast to the traditional methods, deep-learning-based FER shows less dependence on data sets. Moreover, deep-learning-based FER, in contrast to the traditional methods, does not have to consider three major steps (face detection or localization, feature extraction, and classification) separately because the DNN model has the capability to learn a sufficient amount of information to classify seven facial expressions in an end-to-end manner. Among several DNN models, the convolutional neural network (CNN) model is the most popularly employed, especially in the case of static input images. Convolution is a very well-known arithmetic operation in signal processing and image processing when spatial and time-domain data are directly utilized. Before the CNN model was popularly used, frequency domain analysis, e.g., Fourier Transform, was one of the most popular approaches. The CNN model enables direct use of spatial numeric data, i.e., pixel values, for detection, feature extraction, recognition, and classification work. In addition to these, almost all of the fields related to computer vision, image, and signal processing have significant benefits from the CNN model. Usual CNN-based FER takes static images or a set of static images as an input to the network model that is composed of more than three layers, called hidden layers, each of which provides convolution results with the output data of the previous steps. Various structures of filters are convolved with the input data or the output data of the previous layer, leading to an increase in computational complexity which has been resolved with the improvement of hardware infrastructures and the algorithms for developing a light structure of DNN models. Each layer contains the result of the convolution operation providing feature maps followed by generating fully connected layers to proceed to conduct classification. In the recognition work that uses static images as input data, CNN-based approaches have been considered a main method [14]. In practice, recognition tasks in a wild environment may require detection followed by classification in a real-time manner because the input image data varies over time. If DNN models are required to train input face (or facial expression) images with the variation of the expressions over time, i.e., input data has spatiotemporal features, the recurrent neural network (RNN) model is considered more appropriate for the recognition work [15]. In this case, sequences of facial expression data have a temporal dependency in addition to a spatial one, so the additional dependency is taken into account during the classification and recognition process.

**Figure 1:**
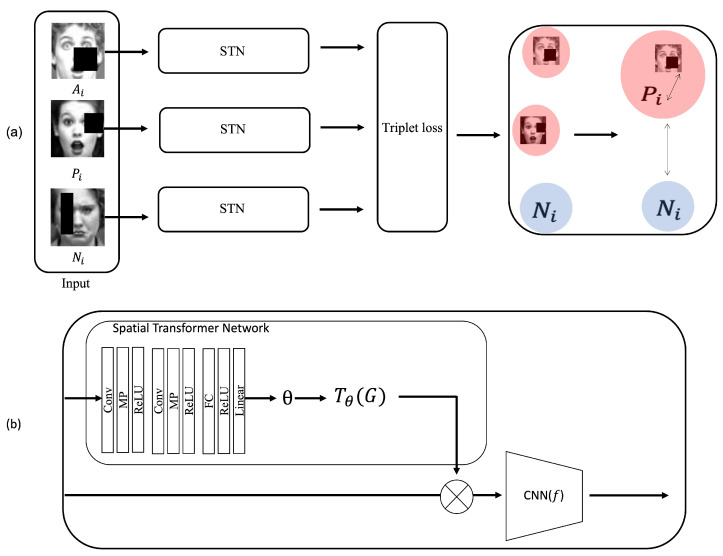
An overview of the proposed model for FER that solves the intra-similarity problem and is robust to occlusion. (**a**) Three classes of facial images with occlusion (anchor, positive and neutral) are classified using the proposed model, STN-TL. (**b**) Architecture of spatial transformer network is basically used in the proposed FER.The images in this figure are from public data set (CK+) [16].

In this paper, we present a novel approach to automatic FER using a spatial transformer network with a triplet loss function. To this end, the proposed method aims at accurate and efficient FER by focusing on the relevant region for each facial expression while robust to occlusion. A flow diagram of the proposed approach is shown in Figure 1.

The rest of this paper is organized as follows. Section 2 briefly introduces related work to the field of FER using deep neural networks and STN. This section also introduces the loss functions that have been applied to the recognition work. In Section 3, we introduce our proposed model TL-STN in detail. Then in Section 4, we introduce the data set used in our experiments. In addition in this section, we describe a comparison between the cross-entropy loss function and triplet loss function and also between occlusion data and non-occlusion data. Then, we compare the state-of-the-art model and TL-STN.

## 2. Related Work

Transformer architecture that has been widely used in the field of natural language processing (NLP) shows exceptionally well-performed results in the recognition task, especially in the case of using sequences of images as input data [17]. Recently, a new learnable module, spatial transformer network (STN) was proposed to provide robust performance by allowing spatial manipulation of input image data. STN is inserted into existing network models, e.g., CNN, and it enables one to achieve robust training results from invariance to the spatial transformation of input data, e.g., translation, rotation, warping, etc. [18]. The STN model has been applied to many practical problems among numerous cases of the recognition problems with encouraging results [19].

Another transformer model, vision transformer (ViT) [20], has gained attention in the recognition field and has been proposed as an alternative to the existing DNN models.

Although the existing FER work has achieved significantly improved results from quantitative and qualitative perspectives, it is still a challenging task due to the existence of uncontrolled external environments, pose variations, or occlusion that degrade the performance of FER results. More complex scenarios of FER need to be dealt with for high-quality FER from practical perspectives. Thus, it is worth investigating FER methods using STN, which has gained attention in the area of deep learning. In this paper, inspired by a spatial transformer network module, FER is performed efficiently by selecting the most relevant part of a facial image followed by applying the triplet loss function. The result is compared to the results using cross-entropy and to the other FER results using state-of-the-art algorithms. The proposed method shows superior recognition results in FER, particularly in the case of facial images having occlusion areas. A spatial transformer network that is included in standard neural network structure has advantages in case of rotation, cropping, scaling, and non-rigid deformation of images that sometimes happen to face images in practice.

As very well-known, traditional methods for the FER employ pixel-level, geometric model, or object-level-based approaches. Recent approaches are usually categorized as deep-learning-based approaches. In the past few years, learning-based approaches have witnessed a significant improvement in recognition tasks, especially in the areas of face recognition, facial expression recognition, activity recognition, etc. In the deep-learning-based approaches, convolutional neural networks and recurrent neural network models are the most widely used. More recently, a transformer network has been considered one of the alternatives to the CNN and RNN-based approaches. In this paper, we are interested in FER using deep-learning-based approaches and the spatial transformer network (STN) with a triplet loss function which improves the success rate of the recognition. The deep neural network (DNN) model enables one to perform automated FER that has long been an interesting and challenging task in the field of recognition problems. Instead of extracting feature points from facial images using a specific mathematical or statistical model, the DNN-based recognition approach extracts diverse and numerous feature points using large numbers of hidden layers that contribute to feature extraction with a brain-like mechanism.

Contrary to the traditional approach that always tries to achieve minimum intra-class distance and maximum inter-class distance in the recognition problem using an analytical model (mathematics, statistics, etc), the recent deep-learning-based approach is able to find abstraction and complex patterns that are inherent in real facial images.

Traditional approaches for the recognition of a face, facial expression, activity, or object lack generalizability due to the variations of a pose or scale and randomly additive noise. In addition, due to the non-existence of data sets, almost all of the recognition task was based on the manual or analytic model-based extraction of feature points. Inspired by the advent of deep learning, CNN-based models have shown robustness to the abovementioned variations, so FER has employed a CNN model to achieve higher accuracy of the recognition rate. CNN-based analysis of facial data has appeared in the work by Lawrence [21], LeCun [22] and Fasel [23] whose work has utilized less than five hidden layers in their network models. Almost all of the FER algorithms also use those works as a baseline to propose novelty or further improvement in the accuracy of the recognition. Since the work of FER in the early stages, significant progress has been achieved in more practical and wild-setting circumstances by utilizing the DNN model. The CNN model is one of the earliest ones that deeply learns and extracts facial feature points that have subtle expression changes that are difficult to extract using traditional recognition methods. Since the introduction of CNN for the recognition work, FER has also employed CNN structure by adding more layers, leading to deep CNN architectures that improved FER results [24]. In the beginning stage of FER using CNN, a limited number of image data sets were used and a specific expression was a target to be recognized. Subject independence and translation, rotation, and scale-invariant FER using CNN has been proposed to discriminate smiling from talking based on the saliency of visual cues [25]. Inspired by the expressions of real emotion, FER has been extended to micro-expression (ME) recognition using deep learning methods [26]. A single deep learning network structure that consists of two convolution layers followed by max pooling and four inception layers was introduced in the early stages of FER using DNN, but this work uses a registered face image data-set and the landmark is extracted a priori [27]. Much research has been conducted to solve wild data set FER problems. The work in [28], proposes a multi-task learning (MTL) framework that exploits the dependencies between these two models using a graph convolutional network (GCN) to recognize facial expressions in the wild. The work in [29], proposes a visual-based end-to-end emotion recognition framework, which consists of the robust pre-trained backbone model and temporal sub-system to model temporal dependencies across many video frames. In addition, facial expressions can be applied in many applications. The work in [30] used facial expression recognition to analyze students’ behavior in the e-learning environment. They used EfficientNet-B2 to extract emotional features in each frame. The sequence of facial images (video sequence) can be used as inputs to the DNN model, and in this case, the temporal relations between frames need to be taken into account, leading to the necessity of a long short-term-memory (LSTM) unit being additionally employed [31]. Another popular deep learning model is the recurrent neural network (RNN) model that is more suitable to temporal, sequential data, such as video, voice, text, etc., leading to the superior performance of the prediction task [32]. Transformer architecture was first proposed as a sequence transduction model based only on attention, and the spatial transformer network (STN) model chiefly deals with images with spatial transformation, so it shows a geometric invariant generalization of differentiable attention that is robust to any spatial transformation. Basic CNN models have the inevitable drawbacks of precise localization of important parts, particularly in the case of small objects. To the best of the authors’ knowledge, there have been fewer research activities on STN-based FER with adaptive loss function. Thus, this section introduces STN-based recognition work (not limited to FER) focusing on the recent literature. The beginning state of deep-learning-based FER could be enhanced by adding an attention mechanism because it can focus on the most important sub-region of a facial image. In FER, the need for an attention mechanism has consequently brought the proposal of the STN model [18]. In [33], the attention mechanism uses a semi-supervised localizer that precisely detects salient regions, and the STN model is inserted into the existing DNN model (e.g., CNN model) to solve the recognition and detection problem in case of spatially transformed input images. The work shows STN incorporates into the basic CNN model, and the whole architecture is composed of three parts, localization, sampling grid, and image sampling. However, it can localize the rough position of the target (e.g., the jersey number of a soccer player), and there is no work on selecting an adaptive loss function for optimization. An attentional convolutional network has been introduced to classify facial expressions where the number of classes is smaller than the usual cases of classification problems [34]. In the work of [34], the authors used less than 10 hidden layers and added an attention mechanism for efficient FER, and the proposed approach reported better accuracy than state-of-the-art results. However, the accuracy shows oscillations, and the work reports that there is a trade-off between the recognition rate and the speed of convergence. Occlusion or pose variations are two major factors that degrade recognition accuracy, so region attention networks (RAN) have been employed for robust FER by adaptively capturing the important facial regions [35]. As FER in the wild is a challenging task, an attention mechanism with a basic CNN model (ACNN) has been proposed to perceive occlusion regions while focusing on the most unoccluded facial regions [36]. In [37], an extension to the basic STN model is proposed by adding procedures for capturing effective attentional regions using facial landmarks or facial visual saliency maps. In [38], STN-based FER was added to the CNN model with spatial and channel attention, and further improvement could be possibly achieved using the proposed GELU (Gaussian error linear unit) activation function. Multimodal emotion recognition that uses speech and facial images has been proposed. In this approach, pre-trained STN for saliency maps and bi-LSTM for the attention mechanism is proposed for emotion recognition [39]. However, in this work, the input image is transformed into mel-spectograms, leading to an increase in computational complexity.

Despite efforts in FER using DNN with an attention mechanism, there is room for further improvement, and our proposed method yields FER that is robust to occlusion and efficient by focusing on the most relevant facial region for specific expression by adding STN. In addition to the methods using STN, our approach employs an adaptive loss function and a triplet loss function that improves recognition accuracy in case of occlusion.

## 3. Proposed Method

**Figure 2:**
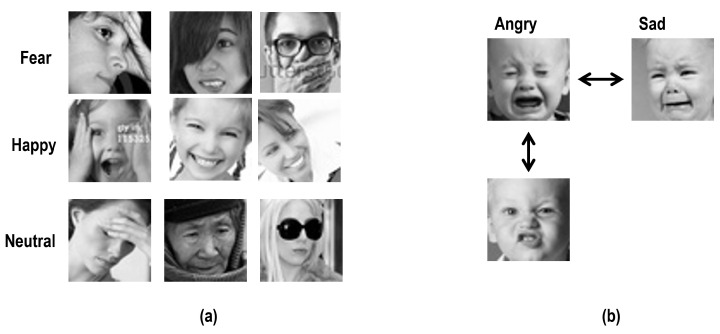
In practice, existing FER algorithms sometimes struggle with intra-similarity problems: (**a**) same expressions from different peoples’ faces, and the existing algorithms sometimes consider them as different expressions; (**b**): different expressions from the same person’s face. Existing work sometimes considers them as the same expressions. The images in this figure are from public data set (FER2013) [40].

Deep-learning-based facial expression recognition research shows high accuracy and performance. Nevertheless, there is still a problem in that it is hard to accurately recognize wild data sets due to external factors such as occlusion, pose, and illumination. Our proposed method is robust to occlusion using STN with an attention mechanism and triplet loss function that achieves optimized recognition accuracy. In practical cases, in addition to the occlusion problem, FER struggles with minimizing intra-class distance, i.e, existing FER algorithms sometimes fail to recognize the same expression. Some examples are depicted in Figure 2a,b.

Different from previous FER images, Figure 2a contains non-formalized FER images. In this case, although the facial images belong to the same class (e.g., fear, happiness, and neutral) the existing FER methods do not successfully classify or recognize the expression. Figure 2b shows another difficult classification problem between “sad” and “angry”. Our proposed method solves this classification problem as well as the occlusion problem using a model called TL-STN which combines the spatial transformer network (STN) and a triplet loss function. In this section, the proposed model TL-STN is briefly explained, and the spatial transformer network and triplet loss are described in detail in Section 3.1 (Figure 1).

### 3.1. Overview of TL-STN

In this section, we introduce an overview of a model that combines a spatial transformer network and triplet loss to solve problems affected by external environments, such as occlusion, pose, and illumination among facial recognition problems. In addition, the proposed method alleviates the limitation of recognizing the same expressions of wild data sets by combining STN and the triplet loss function. In particular, the triplet loss function contributes to aggregation of similar expressions.

As shown in Figure 1a, anchor ($A_{i}$), positive ($P_{i}$), and negative ($N_{i}$) images are used as input data for training the triplet loss function. Anchor data ($A_{i}$) stands for the original data which we want to classify. Positive data stands for the data belonging to the same class as the anchor data. Negative data stands for different class data from the anchor data. In this study, we used three input data for training. Positive and negative data were sequentially picked randomly from the same class and different classes.

Each facial data image is fed to the spatial transformer network followed by a triplet loss function so that the distance between $P_{i}$ and $A_{i}$ is minimized and the distance between $P_{i}$ and $N_{i}$ is maximized. Furthermore, input image ($A_{i},P_{i}$ and $N_{i}$) with occlusion is fed to the STN that is combined with ResNet [41]. To this end, the classification of facial expression with occlusion and under the wild circumstance can be achieved with high accuracy in place of using only deep neural networks (e.g., CNN-based, RNN-based, STN only, etc) with the facial data acquired under the controlled circumstances.

### 3.2. Spatial Transformer Network

When carrying out the classification of facial images based on deep learning technologies, it is important that accurate classification be performed in realistic conditions, such as pose change, occlusion, and missing some parts of the facial image. CNN-based models use a pooling layer to solve these spatial variance problems. The spatial transformer network is a recent classification method [18], which can be utilized in the fields of image classification, co-localization, and spatial attention, and it can solve the spatial invariance problem by transforming specific parts that are required in the learning tasks. Our proposed approach to facial expression recognition combines STN and triplet loss instead of only using a single deep neural network model so that robust recognition can be performed in case of occlusion. In addition to robustness to occlusion, the triplet loss function alleviates the limitation of the existing FER methods that sometimes fail to categorize the same expression as shown in Figure 2. The combination of STN and triplet successfully aggregates the same expression that is captured in wild-set environments.

Figure 1b shows the STN in detail. It consists of a localization network, a grid generator, and a sampler. Conv, MP, ReLU, Linear, and CNN stand for convolution layer, max pooling layer, ReLU activation function, fully connected layer, and CNN model, respectively. ResNet has been used in the STN because it shows the best accuracy of classification in case of occlusion and wild-set environments. The localization network returns parameters required for spatial transformation, and the grid generator returns the grid required for transformation. In the course of transformation, we used affine transformation. The sampler samples the grid and input image generated through the grid generator. The localization network is constructed by adding max pooling and ReLU activation functions with convolution layers and one fully connected layer (Figure 1b).

Through the localization network, six parameters required for the affine grid ($A_{\theta }$), written as Equation (1), are returned as outputs.

(1) $$A_{\theta }=\left[\begin{array}{ccc} \theta_{11} & \theta_{12} & \theta_{13}\\ \theta_{21} & \theta_{22} & \theta_{23} \end{array}\right]$$

The affine grid ($A_{\theta }$) is generated through the six parameters returned from the localization network. The generated grid and input image are sampled through grid sampling to generate a final conversion grid necessary for learning, as written by Equation (2). In Equation (2), $x_{i}$ and $y_{i}$ stand for the coordinates of a horizontal and vertical axis of the generated grid. $T_{\theta }\left(G_{i}\right)$ stands for the grid generator, and $A_{\theta }$ stands for the affine grid.

(2) $$\left(\begin{array}{c} x_{i}\\ y_{i} \end{array}\right)=T_{\theta }\left(G_{i}\right)=A_{\theta }\left(\begin{array}{c} x_{i}\\ y_{i}\\ 1 \end{array}\right)$$

### 3.3. Triplet Loss

FER frequently shows limitations in that different classes (e.g., expressions) of the same person do not show maximized distance, i.e, the existing methods fail to classify different expressions if those are from the same person. FER also shows a limitation in that the existing method fails to aggregate the same expressions of different people. The triplet loss function alleviates this limitation.

Triplet loss is a loss function for metric learning. Based on the anchor data, the triplet loss function enables one to minimize the distance between the same expressions of different people and to maximize the distance between the different expressions of the same person. By using this triplet loss, the Euclidean distance is decreased for facial expression data belonging to the same class, and the Euclidean distance is increased for facial expression data belonging to different classes. Data are organized as follows. The data belonging to the same class as the anchor data are composed of positive data, the data belonging to the other class are composed of negative data, the three sets of data are learned by each STN model, and the output result value is calculated as the Euclidean distance through the triplet loss function, written as

(3) $$\sum_{i}^{N}[||f\left(x_{i}^{a}\right)-f\left(x_{i}^{p}\right){\left|\right|}_{2}-||f\left(x_{i}^{a}\right)-f\left(x_{i}^{n}\right){\left|\right|}_{2}+\alpha ]$$

where *N* is the number of data; *f* is the STN model; *a*, *p*, and *n* are anchor, positive, and negative, respectively, ${\left||.|\right|}_{2}$ means L2 normalization. α is a hyperparameter representing the margin, and in this experiment, it is set to 1.0. In the experiment, back-propagation learning is performed through the above equation for positive data, negative data and anchor data that have passed each STN model.

## 4. Experimental Results

This section details the results with the used data set for the experiments, experimental setup, and environments. To validate the proposed approach in this work, we compare various image data, e.g., occlusion and non-occlusion data. The comparison is carried out through ablation studies, followed by a comparison with the state-of-the-art (SOTA) model.

### 4.1. Data Set

CK+: The Extended CohnKanada (CK+) database [16] is the most widely used laboratory control database in the field of FER. CK+ contains 593 video sequences of 123 topics. The sequences vary in duration from 10 to 60 frames and show transitions from neutral to peak facial expressions. In this video, seven basic facial expression labels (anger, contempt, disgust, fear, happiness, sadness, surprise) are classified based on the FACS (Facial Action Coding System). In this paper, a total of 981 frames were extracted and used in the experiment. Here, 800 training images and 181 test images were randomly divided into experiments.

FER2013: The FER2013 database [40] is the data used in ICML2013 Challenges in Presentation Learning. FER2013 is a large database that is automatically collected by the Google Image Search API. All images are scaled to a size of 48×48 pixels and consist of 7 expression labels (anger, disgust, fear, happiness, sadness, surprise, neutrality). It consists of 28,709 training images, 3589 verification images, and 3589 test images.

#### 4.1.1. Experimental Environment

In this paper, the image size of the data set was adjusted to 224 × 224. Anchor, positive, and negative data were used with batch size eight. Based on the anchor data, images belonging to the same or different classes were randomly extracted to form positive and negative data, respectively. Each of the three data (anchor, positive, negative) is trained through the STN model combined with modified ResNet-18. The triplet loss was calculated through the three output values obtained by the model. We initialized the learning rate to 0.001, and the Adam optimizer was applied. The modified ResNet-18 layers are shown in Table 1. The existing ResNet model is modified by removing the number of layers in the model of ResNet-18, which includes the smallest number of layers among ResNet models. Then, the number of layers becomes smaller.

#### 4.1.2. Ablation Studies

**Figure 3:**
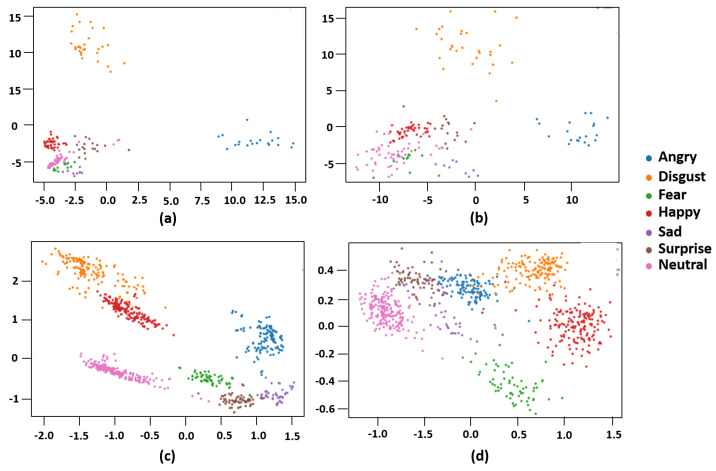
Visualization of data distribution under loss function and occlusion: (**a**,**b**) show classification using the cross-entropy loss function; (**c**,**d**) show the result using triplet loss; (**a**,**c**) visualize the result with non-occlusion data; (**b**,**d**) visualize the result with occlusion data.

Figure 3 visualizes the distribution of image data used for the comparison experiments. The comparison is performed from two perspectives. One is a comparison between occluded facial images and non-occluded ones. The other is a comparison between using cross-entropy and using the triplet loss function. Figure 3a,b represent the case of using the cross-entropy loss function, and (c) and (d) represent the case of using the triplet loss function. Here, (a) and (c) show the visualization of experimental results using occlusion data, and (b) and (d) show the experimental results using non-occlusion data. It seems that it is difficult to clearly distinguish between different classes when a cross-entropy loss is used (Figure 3a,b). On the other hand, when triplet loss is used (Figure 3c,d), it can be seen that classification is more successful. We can see Figure 3a,c show a more clearly distinguished result than (b) and (d) show, which are trained by occlusion data. Nevertheless, when using triplet loss, classes are more clearly distinguished in occlusion data than when using the cross-entropy loss function, and we can see that they are more clearly aggregated between the same classes (indices of vertical and horizontal axes just present relative locations of points of each expression).

Table 2 compares the accuracy of the original ResNet-18 and the modified ResNet-18 using randomly erased CK+ data and compares the accuracy of using cross-entropy loss and triplet loss. Through the experiment, it is confirmed that when the modified ResNet-18 is combined with STN, the accuracy achieved is 2.09% higher than the model combined with STN and the original ResNet-18. Through this experiment, it can be confirmed that when modified ResNet-18 is combined with STN, it shows better performance. In other words, the results imply that the model with fewer layers in the network model shows higher accuracy when combined with STN. In addition, through the experiment, it was confirmed that the use of the triplet loss function showed a classification accuracy of 99.44%, which is 0.48% higher than with the cross-entropy loss. Although our approach uses the ResNet-18 structure, the result is comparable to the result using the ResNet-34 structure (LHC-Net). Unfortunately, our approach struggles with the optimization of the recognition result with an increase in the number of layers. These results verify that in the case of facial expression recognition, using the triplet loss function further improves the accuracy compared to using the cross-entropy loss function.

#### 4.1.3. Comparison Result

Experimental results using CK+ and FER2013 data sets are shown in Figure 3 and Table 2 and Table 3. Table 2 chiefly compares the accuracy between using the cross-entropy and triplet loss function in the ablation study. Table 3 comprehensively shows the comparison results between the SOTA models and our proposed approach. In the case of using the CK+ data set, the proposed model, TL-STN, is compared with FER-IK [42], IPA2LT [43], lp-norm MKL multiclass-SVM [44], twofold random forest classier [45], and the self-supervised learning (SLL) puzzling model [46].

In the CK+ data set, ViT+SE [47] and FAN [48] show high accuracy, but we excluded it from the comparison table because ViT+SE uses 10-fold cross-validation and FAN uses video sequences as input data which is a different setup from our proposed approach. FER-IK is known as a knowledge-augmented image-based FER model, and IPA2LT is known as inconsistent pseudo annotations to the latent truth model. The lp-norm MKL multiclass-SVM is known as multiple kernel learning (MKL) in multiclass support vector machines (SVM). Twofold random forest classier is known as a model which recognizes AUs from image sequences using a twofold random forest classifier. SSL puzzling is known as a nonlinear evaluation in supervised learning (SL) and the self-supervised learning (SSL) puzzling model.

In the case of using the FER 2013 data set, our proposed model, STN with modified ResNet-18 and cross-entropy, is compared to LHC-Net [49], CNN [50], GoogleNet [51], ResNet [41], VGGNet [52], and STN with a cross-entropy loss function.

In the FER2013 data set, Ensemble ResMaskingNet with six other CNNs [40] and Local Learning Deep+BOW [53] showed high accuracy, but we excluded it from the comparison table because these models use machine learning, unlike our proposed model. In addition, simple comparisons are impossible because we propose a loss function using attention-focused mechanism-based models and metric learning.

In this experiment, our approach does not show an improved result, but the proposed model shows superior accuracy compared to the result of using the original ResNet-18 model. TL-STN with the CK+ data set achieves the best recognition accuracy despite using randomly erased facial images.

**Table 3:** Performances comparison with state-of-the-art methods.

Model	Datasets	Accuracy (%)
FER-IK [42]	CK+	97.59
IPA2LT [43]		91.67
lp-norm MKL multiclass-SVM [44]		93.6
Twofold random forest classier [45]		96.38
Nonlinear eval on SL + SSL Puzzling [46]		98.23
**TL-STN (ours)**		**99.41**
LHC-Net [49]	FER2013	74.42
CNN [50]		62.44
GoogleNet [51]		65.20
ResNet [41]		72.4
VGGNet [52]		73.28
**STN (w/orignal ResNet-18) + TL (ours)**		**72.30**
**STN (w/modified ResNet-18) + TL (ours)**		**73.31**

## 5. Conclusions

This paper presents facial expression recognition based on deep learning technology. Since the advent of deep neural network models, diverse applications using image data have shown significant improvement from theoretical and practical perspectives. However, a lot of challenges remain due to the unexpected factors that degrade the performance of recognition. Furthermore, facial expression directly reflects human emotion which is a very qualitative component. Facial expression and human emotion are very delicate, leading to the technical difficulty in analysis and quantification. In this paper, the proposed approach chiefly contributes to two problems, one is occlusion, and the other one is a classification of expression (intra-class similarity problem) in practical cases (Figure 2). Exsiting FER methods usually employ cross-entropy loss function which helps reduce the difference between ground truth values and the estimated (or predicted) ones that are similar to other image recognition fields. The cross-entropy loss function shows a high recognition accuracy for objects that do not change the appearance of objects in the image, but it is difficult to classify when there are various features in the same class, such as facial expressions. In this paper, to solve these problems, the experiments were conducted by using the triplet loss function which was the first suggested in the field of facial expression recognition, and the proposed one can be applied to diverse practical fields. The triplet loss function with a STN (w/modified ResNet-18) alleviates the abovementioned limitations. The proposed model solves occlusion and illumination, poses change issues, and shows superior results to the existing work. To verify the benefit of the modified ResNet-18 model, a comparison was performed that showed a 1.01% improvement on the FER2013 data set. When the triplet loss function and the modified ResNet-18 were combined, they yielded 99.41% accuracy. The experiment with a randomly erased pre-processed CK+ data set showed the highest accuracy compared to SOTA models which were performed with the original CK+ data set. Through these experiments, it was confirmed that even for data with occlusion, our model shows high performance in FER. In addition, the proposed model shows the availability of a metric-learning-based loss function.

In future work, we will more deeply focus on enhancement of the recognition accuracy with the more delicate differences of facial expression, as well as more practical issues in recognition problems. Specifically, we will analyze the practical limitations existing in the proposed approach and will try to solve them through contrastive loss functions such as triplet loss. Through this, we plan to see if we can solve other recognition problems by applying our methods to fine-grain recognition problems that use small data sets, such as medical diagnosis, gender classification, etc. [54,55,56].

## Figures and Tables

**Table 1:** Modified size of each layer of ResNet structure.

Layer Type	Output Size	Patch Size, Channel
Convolution layer 1	24 × 24	7 × 7, 64, stride 2
Convolution layer 2	12 × 12	3 × 3, 64, 3 × 3, 64
Convolution layer 3	6 × 6	3 × 3, 128, 3 × 3, 128
Convolution layer 4	3 × 3	3 × 3, 256, 3 × 3, 256
Convolution layer 5	2 × 2	3 × 3, 512, 3 × 3, 512
Average Pool	1 × 1	-

**Table 2:** Ablation study of ResNet model and using different loss function on CK+ data set.

ResNet	Loss Function	Accuracy (%)
Orignal ResNet-18	CrossEntropy	96.87
Modify ResNet-18	CrossEntropy	98.96
**Modify ResNet-18**	**Triplet**	**99.41**

## Data Availability

The datasets generated during and/or analysed during the current study are publicly available(FER2013 and CK+).
